# 1 plus 1 is more than 2: mental health problems, financial difficulties, and social exclusion in a cross-sectional study of 28,047 general-population adults

**DOI:** 10.1186/s12889-024-18555-1

**Published:** 2024-04-24

**Authors:** Siri Håvås Haugland, Alain Topor, Jan Georg Friesinger

**Affiliations:** Department of Psychosocial Health, University of Agder, Grimstad, Norway

**Keywords:** Mental health, Social exclusion, Socioeconomic status, Poverty, Inequality

## Abstract

**Background:**

Mental health problems and financial difficulties each increase the risk of social exclusion. However, few large studies representing a broad age range have investigated the combined social effect of having both difficulties. The purpose of this cross-sectional study was to examine associations of mental health problems, financial difficulties, and the combination of both with social exclusion.

**Methods:**

This analysis was based on responses from 28,047 adults (age > 18 years) from the general population participating in The Norwegian Counties Public Health Survey 2019. Respondents answered questions about their financial situation, mental health problems, and social exclusion. Social exclusion was measured as a lack of social support, low participation in organized social activities, low participation in other activities, missing someone to be with, feeling excluded, and feeling isolated. Adjustments for sex and age were made in multivariable logistic regression analyses.

**Results:**

Having mental health problems or financial difficulties was associated with various measures of social exclusion (odds ratios [ORs] with 95% confidence intervals [CIs]: 1.33 [1.23–1.43] to 12.63 [10.90–14.64]). However, the odds of social exclusion strongly increased for respondents who reported a combination of mental health problems and financial difficulties compared with those who did not report either (ORs [CIs]: 2.08 [1.90–2.27] to 29.46 [25.32–34.27]).

**Conclusions:**

Having the combination of mental health problems and financial difficulties is strongly associated with increased risk for social exclusion, far beyond the effect of either factor alone.

**Supplementary Information:**

The online version contains supplementary material available at 10.1186/s12889-024-18555-1.

## Background

Social inclusion is a foundation of human health and quality of life, and the World Health Organization (WHO) points to social exclusion as a driving force of health inequalities [1]. The UN Sustainable Development Goal 10.2 [2] targets inequalities between people and groups and emphasizes the social, economic, and political inclusion of everyone irrespective of age, sex, disability, race, ethnicity, origin, religion, or economic or other status. Identifying the factors associated with social exclusion is key to reducing obstacles to social participation and inclusion in communities.

Social exclusion is a multi-dimensional phenomenon, and definitions vary [3]. Most definitions center social relationships and networks [4] and emphasize a lack of participation in social activities [4]. Social exclusion as non-participation also may entail being denied the *opportunity* to participate [4]. In a literature review, the WHO Social Exclusion Knowledge Network [5] discusses the concept of social exclusion and describes it as having three dimensions: structural/economic, contextual/social, and subjective/personal. They further discuss how social exclusion often includes a combination of marginalized economic position and social isolation. Social participation is important to health, but financial resources are often necessary to enable participation and nurturing of social relationships. The more socioeconomic resources a person has, the greater the opportunities to engage in actions that contribute to a healthy life [6], which, in turn, can contribute to social inequalities in health [7–9]. Additionally, people with mental illness may have increased risk of social exclusion [10–12], although some researchers have argued that social exclusion is poorly defined and measured within the mental health literature [4].

It has long been recognized that people with mental illness more often experience a poor economic conditions [7–9]. Living with both mental illness and poverty thus can magnify inequities [7, 13–15] and pose an increased risk for social exclusion. The relationship between low income, mental illness, and social exclusion is complex, but the dominant representation is that social exclusion may be a consequence of mental illness. Thus, the illness can lead to a downward social spiral that ends in poverty, a pattern known as social drift and selection [7]. The directionality of this concept has been challenged by a contradictory representation, known as social causation. In this framework, the process starts with the challenges associated with living in poverty, characterized by economic strain with poor housing, work, and educational conditions and violence and insecurity [16]. These factors in turn drive behaviors such as the tendency to withdraw from social interaction [17], including social interaction with family and friends and from other forms of community involvement and participation [18]. However, studies regarding the causal direction between mental health problems and poverty’s different aspects are not conclusive [19]. Kirkbride et al. [16] argue for a “bi-directional” relationship, even if they argument for social preventive measures in their article. This withdrawal is often interpreted outside of the social context as a symptom of a mental illness. This social causation representation problematizes the concept of mental illness. As Cohen [20] wrote, “Terms used in descriptions of the poor, including apathy, resignation, low self-esteem, alienation, and distrust of others, are also commonly used to describe chronic mental patients” (p. 954). In this interpretation, behaviors that are described in psychiatric diagnostic manuals, as symptoms of an illnesscould be understood as arising from a life lived within the restrictions caused by poverty Placed in their social context, these behaviors could be defined as reasonable reactions to a scarcity of resources needed to live a decent life [7, 21, 22], Without the financial ability to participate in social activities, opportunities to feel valued or add value to other people also may be reduced, leading to feelings of not mattering, which again in turn are associated with loneliness and mental health problems [23, 24].

Studies investigating mental health and social exclusion often focus on selected populations such as people with severe mental illness, specific age groups, or smaller numbers of patients [3]. Some studies also interchangeably use indicators of poverty and social exclusion,, making it difficult to interpret which indicators primarily measure financial difficulties or social exclusion [4]. To our knowledge, there are limited large-scale studies encompassing a wide age range from the general population that have investigated the combined impact of mental health issues and financial difficulties on social life and participation. The purpose of this study was therefore to investigate the associations between having mental health problems, financial difficulties, or both, and the experience of social exclusion.

## Materials and methods

### Norwegian counties public health survey

**Fig. 1:**
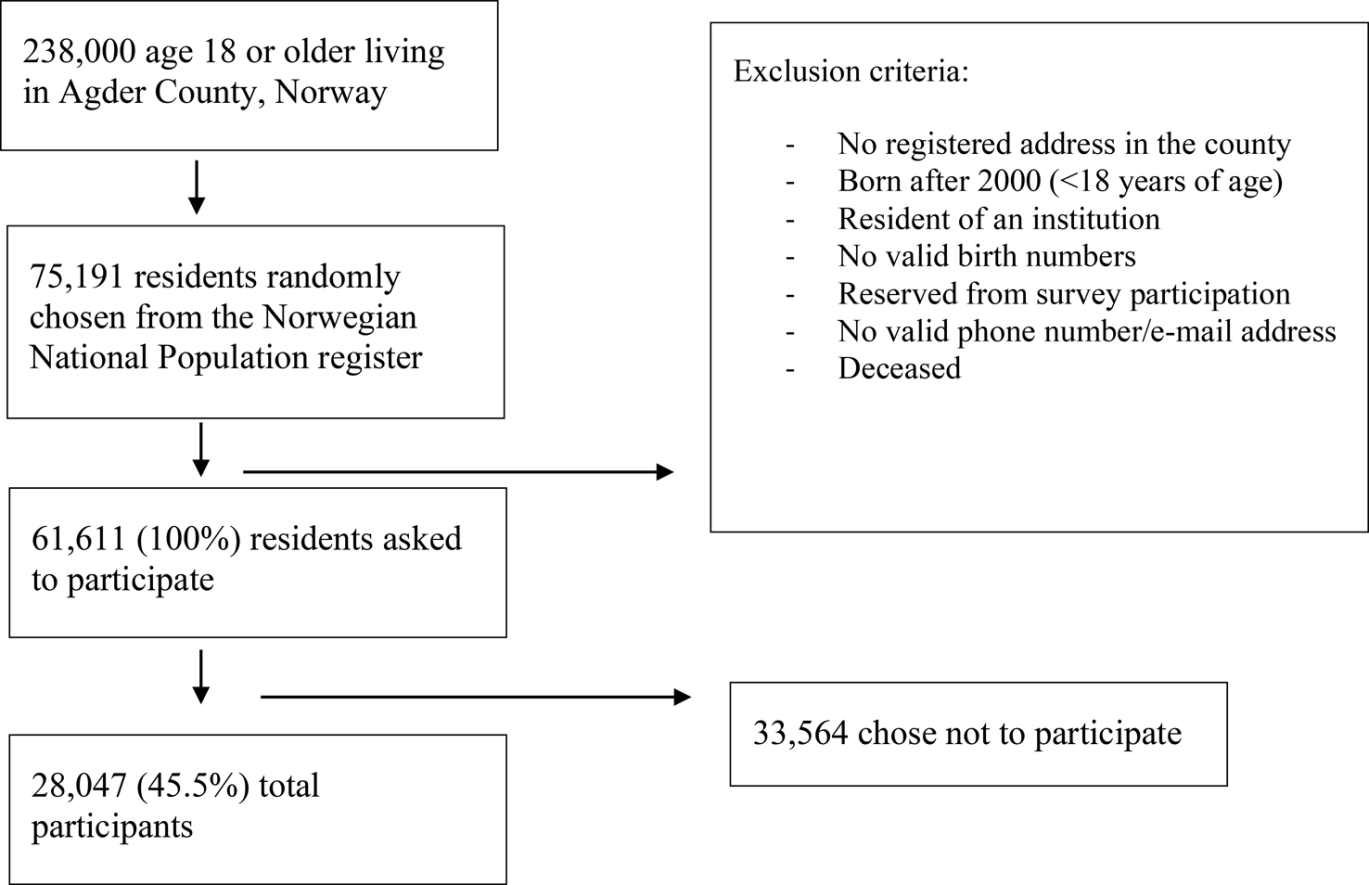
Flow chart of study criteria – The Norwegian Counties Public Health Survey, conducted in Agder, 2019

The Norwegian Counties Public Health Survey was conducted in 2019 and consists of cross-sectional data on health, well-being, childhood, living conditions, local environments, accidents, and injuries among adults aged 18 or older. A total of 75,191 (31.6%) people in Agder County were randomly selected from the Norwegian Population Registry of inhabitants in Southern Norway. E-mails or telephone numbers were obtained from the contact registry of the Norwegian Agency for Public Management and eGovernment. After exclusions based on specified criteria (see Fig. 1), invitations to participate in the online health survey were sent by e-mail and text messages. Participants provided consent by completing the online questionnaire, which took approximately 15 min. A total of 28,047 county inhabitants participated (response rate 45.5%; 14,925 [53%] women). Young adults had a lower response rate, especially among men aged 18–39 years. In addition, participants with higher education are overrepresented.

Table 1 shows the questions, response categories, and definitions used in the current study. The English translation of the questionnaire can be found in the supplemental material.

**Table 1:** Norwegian Counties Public Health Survey, conducted in Agder County, 2019

Variable	Questions	Response options	Definitions
Mental health problem/ distress	HSCL-5. The HSCL-5 is a five-item short version of the Hopkins Symptom Checklist (HSCL-25) and has been found reliable and valid as a screening instrument for symptoms of depression and anxiety.	Response categories were ‘not bothered’, ‘a little bothered’, ‘bothered quite a lot’, and ‘extremely bothered’.	The total HSCL-5 score was summed, and a cut-off point at > 2.0 was used to define mental health problems.
	The participants were asked if over the last week, they had: 1) been constantly afraid and anxious 2) felt tense or keyed up 3) felt hopeless about the future 4) sad or depressed 5) worried		
Financial difficulties	For one-person households, consider your total income. If you live with others, consider the total income of everyone in the household. How easy or difficult is it for you to make ends meet day to day with this income?	1. Very difficult 2. Difficult 3. Relatively difficult 4. Relatively easy 5. Easy 6. Very easy 7. Don’t know	1–3 = financial difficulties vs. 4–7 = no financial difficulties
Social exclusion measured with 6 separate measures	**Oslo Support Scale** Social support was measured by Oslo Support Scale, which is a common instrument widely used to assess level of social support.		The three variables were summed and then dichotomized. Respondents with a sum score of 3 to 8 were defined as having low social support.
	Oslo 1: How many people are so close to you that you can count on them if you have great personal problems?	Oslo 1: ‘none’ ‘1–2’ ‘3–5’ ‘5+’	
	Oslo 2: How much interest and concern do people show in what you do?	Oslo 2: 1 ‘none’ 2 ‘little’ 3 ‘uncertain’ 4 ‘some’ 5 ‘a lot’	
	Oslo 3: How easy is it to get practical help from neighbors if you should need it?	Oslo 3: 1 ‘very difficult’ 2 ‘difficult’ 3 ‘possible’ 4 ‘easy’ 5 ‘very easy’	
	How often do you participate in organized activity/voluntary work such as sports team, political organization, religious communities, choir, or similar?	1. Daily 2. Weekly 3. 1–3 times a month	4–5 = low participation 1–3 = regular participation/ref group
	How often do you participate in other activities such as a club, meetings, time with friends, exercise with friends/colleagues, or other?	4. Seldom 5. Never	
	How often do you feel you miss someone to be with?	1. Never 2. Seldom 3. Sometimes 4. Often 5. Very often	4–5 = missing someone to be with 1–3 = reference group
	How often do you feel excluded?	1. Never 2. Seldom 3. Sometimes 4. Often 5. Very often	4–5 = excluded 1–3 = not excluded/reference group
	How often do you feel isolated from others?	1. Never 2. Seldom 3. Sometimes 4. Often 5. Very often	4–5 = isolated 1–3 = not isolated/reference group
Sex	Sex	Retrieved from registries	Male/female
Age	Age in years	Retrieved from registries	Age applied as a continuous variable (year)

### Data analysis

Initial descriptive analyses were performed to provide an overview of participant characteristics (Table 2). Additionally, multivariable logistic regression was conducted (Table 3) separately for each of the exposure variables (mental health problems and financial difficulties), as well as jointly, with six indicators of social exclusion as dependent variables. These indicators were dichotomized from scale responses (Table 1).

**Table 2:** Descriptive statistics

	n	%
Male	13,122	46.8
Female	14,925	53.2
Mental health problems, but not financial difficulties	3516	15.7
Financial difficulties, but not mental health problems	3039	14.5
Both mental health problems and financial difficulties	2489	9.5
Lack of social support	3023	10.8
Seldom/never participation in organized social activities	14,541	52.0
Seldom/never participation in other social activities	4638	16.6
Often/very often miss someone to be with	2767	9.9
Often/very often feel excluded	2266	8.1
Often/very often feel isolated	1945	6.9

**Table 3:** Multivariable logistic regression of associations between having mental health problems or having financial difficulties or both, and indicators of social exclusion

	Lack of social support	Low participation in organized social activities	Low participation in other social activities	Miss someone to be with	Feel excluded	Feel isolated
	OR (95% CI)	OR (95% CI)	OR (95% CI)	OR (95% CI)	OR (95% CI)	OR (95% CI)
Model 1: Mental health problems, but not financial difficulties	5.10 (4.57–5.68)***	1.60 (1.48–1.72)***	1.97 (1.79–2.16)***	6.22 (5.56–6.95)***	11.73 (10.28–13.38)***	12.53 (10.83–14.51)***
Model 2: Financial difficulties, but not mental health problems	3.05 (2.68–3.47)***	1.33 (1.23–1.44)***	1.66 (1.50–1.84)***	2.72 (2.37–3.14)***	3.00 (2.51–3.60)***	3.41 (2.79–4.16)***
Model 3: Both mental health problems and financial difficulties	13.21 (11.82–14.77)***	2.08 (1.90–2.27)***	3.39 (3.06–3.75)***	13.49 (12.02–15.14)***	26.84 (23.43–30.74)***	29.46 (25.32–34.27)***

Data are presented as odds ratios (ORs) with 95% confidence intervals (CIs). * *p* < 0.05, *** *p* < 0.001. Results are adjusted for age and sex

The reference group in all three models was participants who reported neither mental health problems or financial difficulties

The first analysis compared the six indicators of social exclusion between individuals who reported mental health problems without financial difficulties and those who did not report either. The second analysis examined the six social exclusion indicators between individuals who reported financial difficulties without mental health problems and those who reported neither. Lastly, the third analysis compared the six indicators between individuals who reported both mental health and financial difficulties and those who reported neither. All three analyses included adjustment for sex and age. Results are reported as adjusted odds ratios (ORs) with 95% confidence intervals (CIs) and the level of statistical significance set to 5%. All analyses were conducted using IBM SPSS Statistics 28.0.

## Results

### Descriptive analysis

The participant age range was 18–94 years, and the mean age was 46.9 years (standard deviation 16.30).

**Fig. 2:**
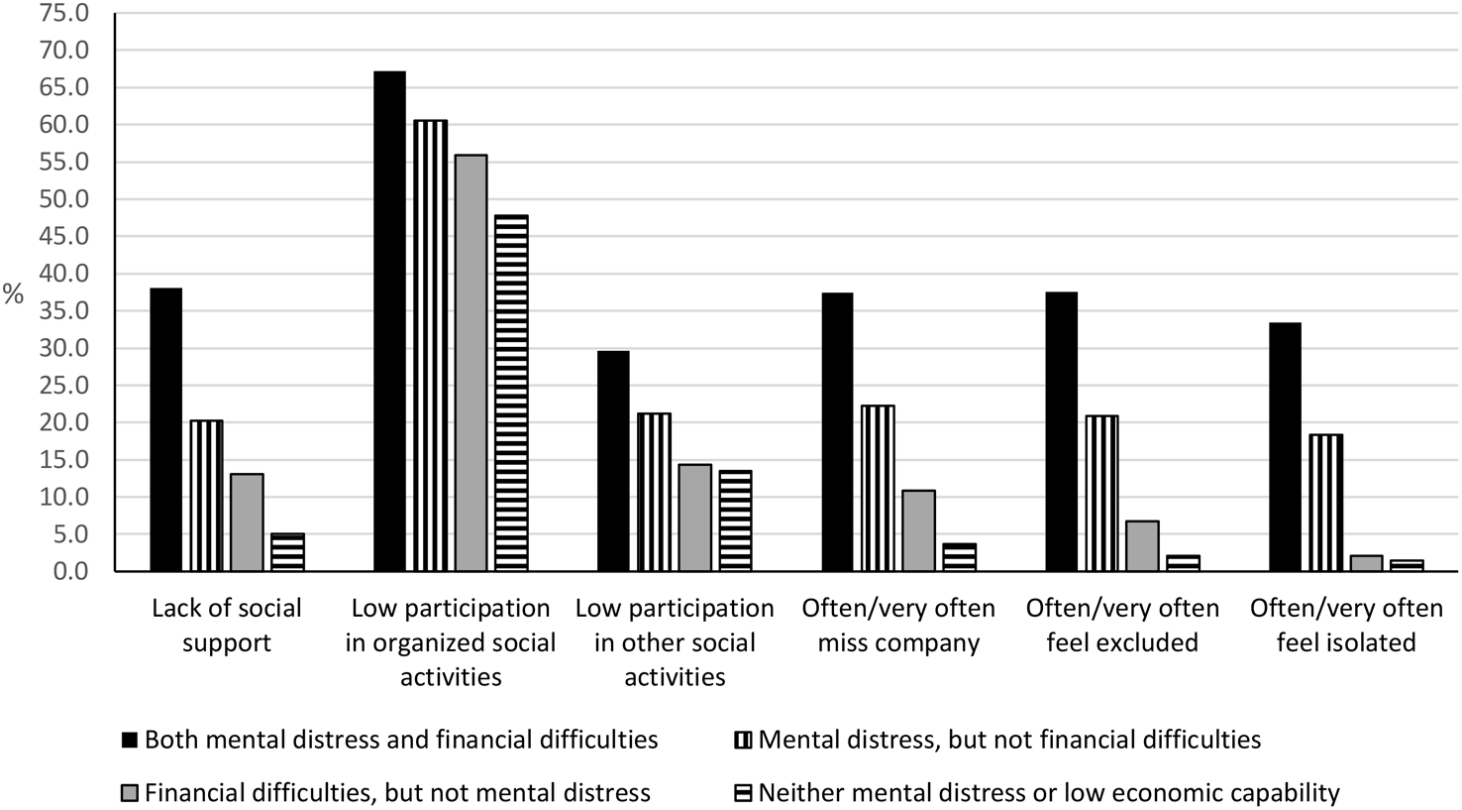
The proportion of people experiencing social exclusion in relation to financial difficulties and/or mental distress

Figure 2 illustrates the consistent pattern of social exclusion indicators being less common among those without mental health problems and financial difficulties compared with the other groups. Among those with financial difficulties and no mental health problems, slightly more people experienced indicators of social exclusion, followed by those with mental distress and no financial difficulties. A steep increase in the proportion reporting various types of social exclusion indicators was seen among respondents citing both mental distress and financial difficulties.

### Multivariable logistic regression results

Multivariable logistic regression adjusted for age and sex showed that compared with respondents reporting neither mental health problems nor financial difficulties, mental health problems alone (OR 5.10; CI 4.57–5.68) and financial difficulties alone (OR 3.05; CI 2.68–3.47) were strongly associated with a lack of social support. Mental health problems alone (OR 6.22; CI 5.56–6.95) and financial difficulties alone (OR 2.72; CI 2.37–3.14) also were associated specifically with missing someone to be with. Those who reported having the combination of mental health problems and financial difficulties had significantly higher odds of also reporting a lack of social support (OR 13.21; CI 11.82–13.77) or missing someone to be with (OR 13.49; CI 12.02–15.14) compared with those reporting neither.

Across all six indicators, those who reported mental health problems, financial difficulties, or both consistently had increased odds of social exclusion. Compared with reporting no mental health problems, reporting mental health problems alone was strongly associated with feeling excluded (OR 11.73; CI 10.28–13.38) or isolated (OR 12.53; CI 10.83–14.51). Having financial difficulties alone also at least tripled the odds of feeling excluded (OR 3.00; CI 2.52–3.60) or isolated (OR 3.41; CI 2.79–4.16), although the risk increase was not as steep as with mental health problems. Reporting both mental health problems and financial difficulties was strongly associated with feeling excluded (OR 26.84; CI 23.43–30.74) or isolated (OR 29.46; CI 25.32–34.27) [23].

## Discussion

In this study, we investigated the associations between social exclusion and having mental health problems or financial difficulties or both. Social exclusion was more common among those who reported mental health problems or financial difficulties compared with those who reported neither. As discussed in the introduction, mental health and financial difficulties may be interrelated and act as mutual reinforcers, which led us to assess social exclusion among those who reported having both life difficulties. Our findings showed that the odds of social exclusion were highest among respondents having both mental health problems and financial difficulties compared with those reporting neither. This risk of social exclusion when enduring the combination of mental health problems and financial difficulties increased far beyond the effect of each factor alone, suggesting a synergism beyond “1 + 1 = 2” and identifying a group at very high risk for social exclusion.

Unraveling the intricate interplay of processes associated with financial difficulties, mental health problems, and social exclusion is challenging, and our findings do not provide a comprehensive understanding of this complexity. However, we find it useful to discuss possible perspectives on how these life difficulties might inter-relate. Social exclusion cannot be explained simply by individual characteristics such as mental health problems or socioeconomic status and must be seen as interactions between individuals and their opportunities for social participation.

Mental health problems and financial difficulties can impact the development of social relationships. Chronic or long-term mental health problems may result in a lack of social capital and resources, which hinder individuals’ ability to establish connections with others [12]. Without the funds to take the bus to a café to meet a friend or to assist in practical situations, developing and maintaining social relationships is difficult [25]. In a study of reciprocity and fragmentation of social life, Offer [18] highlights the bidirectional process of social isolation and withdrawal related to poverty. The experience of poverty can lead to individuals withdrawing from social life due to both economic constraints and the stress of relying on others without being able to reciprocate. For the same reason, the poor person might also be excluded from social life, as relationships often include reciprocity. In this case, it is the social network, not the person, that withdraws from the relationship and does the excluding. Thus, being unable participate in this reciprocity could reinforce a sense of worthlessness in persons with mental health problems or financial difficulties, of not mattering and being unable to participate in social relations. This relationship is supported by the results of supplemental analyses (not shown) of how respondents assessed how much they matter to others. Compared with those citing no mental health or financial difficulties, respondents with both had a significantly lower score on the question of whether they actively contribute to the happiness and well-being of others. Thus, financial difficulties or mental health problems may reinforce obstacles to handling the reciprocal and transactional nature of mattering [26]. Reciprocal mattering includes both having and giving value to others [27], which presupposes opportunities of agency that may be inaccessible if economic resources are scarce or mental problems hinder participation.

The combination of mental health problems and financial difficulties characterizes a group that is particularly vulnerable to social exclusion beyond the risk associated with each factor alone. This synergy could suggest that financial difficulties and mental health problems should be addressed together to prevent social exclusion. However, given the interrelations among mental health, finances, and social exclusion, a change in one area may lead to change in another. Most of the recovery literature stresses the importance of breaking social marginalization and creating social relationships and community involvement [12]. These steps might be facilitated by an improved economic situation that enables sufficient housing and clothing to allow for treating and entertaining friends and family. Indeed, in studies of “supported socialization” for persons with “severe mental illness,” those given extra money experienced an improvement in their mental state and social contacts [13, 14, 28]. Our findings also can be seen in the light of Tew’s concept of recovery capital [26], which differentiates among five forms of capital: economic, social, relational, identity, and personal. Tew [26] stresses that access to one form of capital is not enough, implying that the combination of the different capitals constitutes the basis for a recovery journey. Social work practitioners commonly assess individuals’ access to different forms of capital and work towards enhancing their resources collectively [29]. However, many practitioners understand this process as implying only a cognitive change in the person’s “values, feelings, goals, skills, and/or roles” [27] and not change in their real-life conditions.

Our findings are based on data from the general population and should be relevant for broader strategies and policies entailing a greater awareness of social exclusion. The risk of social exclusion in this Norwegian population sample may be unexpected with regard to the basic aims of Nordic welfare arrangements to reduce (health) inequalities and improve participation for all citizens [30, 31]. Scholars have called this phenomenon the “Nordic paradox” because of the persisting inequalities in health and mortality [32, 33]. Our findings appear to reflect this paradox but need to be seen in the context of a growing wealth inequality globally. Poverty – or more precisely, relative poverty– has again become a public health concern in Western countries, affecting people’s mental states and societal participation [8, 32–34]. One result might be increased social exclusion of those who are disadvantaged, which also could affect their mental health.

Our study emphasized social exclusion risks for people who had both mental health and financial challenges, indicating a severe downward pressure on any recourse. According to Tew [35], the recovery capitals of these marginalized groups are low, and welfare politics therefore must focus on policies that increase capitals in these overlooked populations. Another implication at the community level could be the need for places that facilitate mattering and participation [36]. Webber and Fendt-Newlin [36] identified a range of approaches (individual social skills training, group skills training, supported community engagement, group-based community activities, employment interventions, peer support interventions) aimed at strengthening social participation for people with mental health problems. Although some interventions offered promising outcomes, the evidence of what works is limited [36].

That leaves the question of where to start and what to do. While our study does not propose specific measures, it does make a valuable contribution by highlighting the strong association between experiencing multiple life difficulties, such as mental health problems and financial hardships, and an increased risk of social exclusion. By emphasizing this relationship, our findings underscore the importance of addressing these interconnected challenges to mitigate the risk of social exclusion. Kirkbride et al. [16] have emphasized the importance of intervening on modifiable social determinants that have an impact on mental health problems and suggested that prevention of social exclusion should start with social determinants to secure adequate living conditions and the economic foundation for social participation. Priebe [37] goes even further and writes (p. 1): “What should be done? Obviously, in order to achieve substantial improvements in public mental health, we require societies to change and implement all those factors that promote mental health.” Among the factors Priebe mentions is that “societies should provide safe and supportive upbringing conditions; eradicate poverty; promote social cohesion and functional communities; and have little social inequality.” He states (p. 1) that “These requirements are clear and unequivocal, no more research needed.”

## Strengths and limitations

A clear strength of this study is the large number of participants with a broad age range drawn randomly from a general population. However, the participants included a greater proportion of adults with higher education compared with the total adult population in Norway. This skew may have increased the risk of bias in analyses related to economic capability and perhaps have led to an underestimation if people with financial difficulties were underrepresented. While the response rate was comparatively lower than what is typically observed in school-based surveys or similar studies, it was still deemed satisfactory when compared to other online surveys [38], However young adults, particularly men aged 18–39 years, exhibited a lower response rate, indicating a challenge in engaging this demographic group effectively.

The cross-sectional design limits drawing any inferences about causality related to the identified associations, and the results should be interpreted with caution. As mentioned in the introduction, previous studies have indicated that relationships between mental health and social exclusion can be bi-directional. For instance, social exclusion can impact mental health outcomes, and vice versa.

Further, the findings rely on self-reported data, which can be influenced by social desirability bias and recall bias. While self-report data is widely used in surveys, it is important to acknowledge that the combination of self-reported data and a cross-sectional design has raised concerns and limitations in research [39]. We addressed social desirability by utilizing a validated self-report instrument, specifically the HSCL-5, to assess mental health problems. Self-reported data can be appropriate for capturing subjective perspectives, such as participants’ perceptions of their financial situation. To minimize recall bias, we asked participants about symptoms of mental health problems within the past week and their current financial situation. We also acknowledge the possibility that unmeasured factors, such as cultural influences or neighborhood socioeconomic status, may have influenced individuals’ perceptions of what qualifies as financial difficulties or mental health problems. These unaccounted factors could potentially act as confounders, influencing the associations observed in our study. Low participation in organized activities was quite common among our respondents, and more than half reported that they never or seldom participated in organized activities. This particular measure may therefore not be especially suitable as a marker of social exclusion.

## Conclusion

The association between having both mental health problems and financial difficulties and feeling excluded or isolated was very strong and suggests a cumulative effect beyond “1 + 1 = 2.” Efforts to prevent social exclusion should include measures to secure the financial resources needed to participate socially.

### Electronic supplementary material

Below is the link to the electronic supplementary material.

Supplementary Material 1

## Data Availability

The data that support the findings of this study are available from Norwegian Institute of Public Health, but restrictions apply to the availability of these data, which were used under license for the current study, and so are not publicly available. The dataset supporting the conclusions of this article is available in the repository Helsedata (https://helsedata.no/en/) upon application to Norwegian Institute of Public Health.
