# Supplementary material for: 1 plus 1 is more than 2: mental health problems, financial difficulties, and social exclusion in a cross-sectional study of 28,047 general-population adults
Source: BMC Public Health. 2024 Apr 24;24:1148. doi: 10.1186/s12889-024-18555-1 (PMC11044315; doi:10.1186/s12889-024-18555-1)
Supplement: Supplementary file 1 — Supplementary Material 1 [file 12889_2024_18555_MOESM1_ESM.pdf]

Translation of information related to consent and questionnaire

Consent

What happens to your information?

The consent applies to the county health survey in Vest-Agder and Aust-Agder 2019.

The main purpose of the survey is to obtain results that your county and municipality of residence can use in public health work. The survey involves you completing a questionnaire about health, health behavior, well-being and quality of life.

The collected information will also be used for research that can provide more knowledge about the population's health and factors that affect health. In this context, it may be relevant to obtain information from other registers as well, for example information about education, income and social security benefits from registers managed by Statistics Norway, or information about illness and treatment from medical registers such as the Cancer Registry, Norwegian Patient Register, Cardiovascular Disease Register. Obtaining additional information is also necessary to get the best possible analyses and perform quality control.

Information from the county health survey in Vest-Agder and Aust-Agder may also be included in a combined county health survey for the entire country. It may also become relevant to compile information from the county health survey in Vest-Agder and Aust-Agder with other health surveys, such as the Norwegian Mother, Father and Child Cohort Study.

The information you provide in the questionnaire will be stored and processed in accordance with current privacy regulations. All information will be processed without name and national identity number or other directly identifying information. The national identity number is stored separately from other information about you.

All research will follow the rules in the Health Research Act and be carried out by the Norwegian Institute of Public Health or other research institutions approved by regional research ethics committees.

Results will only be published for groups and never at the individual level. This means that results based on responses from only one or very few individuals will not be published.

The data collection is authorized in the Regulation on overview of public health § 7. In order to facilitate statistics and research, the information will be stored indefinitely after the collection is completed. The purpose is to follow the development of health and living conditions over time and, among other things, investigate the causes of disease. In any follow-up studies, you may be contacted again.

After the data collection is completed, the county municipality/municipality of residence will be sent anonymous data files for further analysis. All personal identifying characteristics and information that can be used to identify participants will be removed from the data files prior to submission.

You will not receive individual feedback on results from the county health survey.

You have the right to access what information is stored about you and what the information has been used for.

It is voluntary to participate, and you can withdraw your consent at any time and request that the information be deleted. You can do this by sending an email to fylkeshelseundersokelser@fhi.no

The Norwegian Institute of Public Health is responsible for the survey. The Data Protection Officer at the institute is Erlend Bakken.

Information about your rights can be found on the Norwegian Institute of Public Health's website: www.fhi.no/studier/fhus/ Here you will also find contact information for the person responsible for processing, the person responsible for research, and the Norwegian Institute of Public Health's Data Protection Officer.

Results from the survey will be published on the county's websites. Research based on the survey will be published on the Norwegian Institute of Public Health's websites www.fhi.no/studier/fhus/

If you wish to complain about the processing of your information, you can send a complaint to the Norwegian Data Protection Authority.

I consent/approve that the information I have provided can be used in accordance with the above. *

-Yes

Questionnaire

Neighborhood and Community

1. To what extent do you feel at home in your local area?

- To a great extent
- To some extent
- To a small extent
- Not at all

2. To what extent do you feel safe in your local area?

- To a great extent
- To some extent
- To a small extent
- Not at all

Access to Local Facilities and Services

3.1. Do you find cultural and sports facilities (cinema, library, swimming pool, sports hall, etc.) easily accessible?

- Very good accessibility
- Good accessibility
- Neither good nor poor accessibility
- Poor accessibility
- Very poor accessibility
- Don't know

3.2. Do you find shops, restaurants and other services easily accessible?

- Very good accessibility
- Good accessibility
- Neither good nor poor accessibility
- Poor accessibility
- Very poor accessibility
- Don't know

3.3. Do you find public transport easily accessible?

- Very good accessibility
- Good accessibility
- Neither good nor poor accessibility
- Poor accessibility
- Very poor accessibility
- Don't know

3.4. Do you find it easy to access nature and outdoor recreation areas?

- Very good accessibility
- Good accessibility
- Neither good nor poor accessibility
- Poor accessibility
- Very poor accessibility
- Don't know

3.5. Do you find parks and other green areas easily accessible?

- Very good accessibility
- Good accessibility
- Neither good nor poor accessibility
- Poor accessibility
- Very poor accessibility
- Don't know

3.6. Do you find that pedestrian and cycle paths are well developed in your local area?

- Very good
- Good
- Neither good nor poor
- Poor
- Very poor
- Don't know

Participation in Activities

4. How often do you participate in organized activities/voluntary work such as sports clubs, political associations, religious communities, choirs or similar?

- Daily
- Weekly
- 1-3 times per month
- Less frequently
- Never

5. How often do you participate in other activities, such as clubs, meetings, meeting friends, exercise walks with friends/colleagues or others?

- Daily
- Weekly
- 1-3 times per month
- Less frequently
- Never

Health

6. How would you rate your overall health? Would you say it is...

- Very good
- Good
- Neither good nor poor
- Poor
- Very poor

7. How would you rate your dental health? Would you say it is...

- Very good
- Good
- Neither good nor poor
- Poor
- Very poor

8. When was the last time you visited a dentist/dental hygienist?

- 0-2 years ago
- 3-5 years ago

- More than 5 years ago

8a. If more than 5 years since your last dentist visit, why? (Select one or more)

- Financial reasons
- Fear/anxiety
- Transportation issues
- Other reasons

9. All in all, when you think about how you are doing these days, are you mostly satisfied with your life or mostly dissatisfied?

- Very satisfied
- Quite satisfied
- Neutral
- Quite dissatisfied
- Very dissatisfied

Body Height and Weight

10. What is your height without shoes? (Enter in cm)

11. What is your weight without clothes and shoes? (Enter in whole kg. If pregnant, weight before pregnancy)

Mental Health Issues

12.1. To what extent have you been bothered by nervousness or inner unrest in the last week?

- Not bothered
- A little bothered
- Quite bothered
- Very bothered

12.2. To what extent have you been bothered by fear or anxiety in the last week?

- Not bothered
- A little bothered
- Quite bothered
- Very bothered

12.3. To what extent have you been bothered by a feeling of hopelessness about the future in the last week?

- Not bothered
- A little bothered
- Quite bothered
- Very bothered

12.4. To what extent have you been bothered by depression or melancholy in the last week?

- Not bothered
- A little bothered
- Quite bothered
- Very bothered

12.5. To what extent have you been bothered by worry or anxiety in the last week?

- Not bothered
- A little bothered
- Quite bothered
- Very bothered

12.6. To what extent have you been bothered by sleep problems in the last week?

- Not bothered
- A little bothered
- Quite bothered
- Very bothered

Long-term Health Problems and Disabilities

13. Do you have any long-term illnesses or health problems? We also mean illnesses or problems that are seasonal, or that come and go. The condition is that it/they have lasted, or are expected to last at least six months.

- Yes
- No

13a. If yes, how do these health problems affect your daily life?

- To a great extent
- To some extent
- To a small extent

- Not at all

14. Do you have any disability or do you suffer from problems due to injury? We also mean problems that come and go.

- Yes
- No

14a. If yes, how do these disabilities affect your daily life?

- To a great extent
- To some extent
- To a small extent
- Not at all

Social Support and Loneliness

Social Support

15.1. How many people are so close to you that you can count on them if you have major personal problems? Also include closest family.

- None
- 1-2
- 3-5
- 6 or more

15.2. How much interest do others show in what you do? Would you say they show...

- Great interest
- Some interest
- Neither great nor little interest
- Little interest
- No interest

15.3. Getting practical help from neighbors if you needed it, is it...

- Very easy
- Easy
- Neither easy nor difficult
- Difficult
- Very difficult

Loneliness

16.1. How often do you feel that you miss having someone to be with?

- Never
- Rarely
- Sometimes
- Often
- Very often

16.2. How often do you feel left out?

- Never
- Rarely
- Sometimes
- Often
- Very often

16.3. How often do you feel isolated from others?

- Never
- Rarely
- Sometimes
- Often
- Very often

Health related behavior

Physical activity

17. Approximately how many hours do you spend sitting still on a typical weekday? Include time spent at work, school, and leisure activities. Provide the number of whole hours.

18. How often do you usually exercise or engage in physical activity during your free time? (Take an average)
By physical activity, we mean activities such as walking, skiing, swimming, or participating in sports.

- Never
- Less than once a week
- Once a week
- 2-3 times a week
- 4-5 times a week
- Almost every day

(The following 18a and 18b items appears if any of the following options are selected for question "18. How often do you usually exercise or engage in physical activity during your free time? (Take an average)": Once a week, 2-3 times a week, 4-5 times a week, Almost every day, Less than once a week)

18a. How intense is your exercise or physical activity? (Take an average)

- I take it easy without getting out of breath or sweaty
- I exert myself enough to get out of breath and sweaty
- I push myself almost to my limits

18b. How long do you exercise each time? (Take an average)

- Less than 15 minutes
- 15 minutes - 29 minutes
- 30 minutes - 1 hour
- More than 1 hour

Diet

19.1. How often do you usually drink sugary soda or soft drinks?

- Rarely/Never
- 1-3 times per month
- Once per week
- 2-3 times per week
- 4-6 times per week
- Daily

19.2. How often do you usually eat fruits and berries (excluding juice or fruit juice)?

- Rarely/Never
- 1-3 times per month
- Once per week
- 2-3 times per week
- 4-6 times per week
- Daily

19.3. How often do you usually eat vegetables (including salad)? Exclude potatoes.

- Rarely/Never
- 1-3 times per month
- Once per week
- 2-3 times per week
- 4-6 times per week
- Daily

19.4. How often do you usually eat fish (as a topping, for lunch or dinner)?

- Rarely/Never
- 1-3 times per month
- Once per week
- 2-3 times per week
- 4-6 times per week
- Daily

Tobacco

20. How often do you smoke?

- Daily
- Occasionally
- Not now, but previously daily
- Not now, but previously occasionally
- Never smoked

21. How often do you use snus/snuff?

- Daily
- Occasionally
- Not now, but previously daily
- Not now, but previously occasionally
- Never used snus/snuff

Alcohol

Now we move on to questions about alcohol. Here we refer to all alcoholic beverages such as beer, wine, spirits, alcopops, and so on.

22. Have you ever consumed alcohol?
    Yes
    No

(The following item appears if the following option is selected for question "22. Have you ever consumed alcohol?": Yes)

22a. In the past 12 months, how often have you consumed alcohol?

- Never
- Monthly or less
- 2-4 times per month
- 2-3 times per week
- 4 or more times per week

(The following (22 b and c) items appears if any of the following options are selected for question "22a. In the past 12 months, how often have you consumed alcohol?": Monthly or less, 2-4 times per month, 2-3 times per week, 4 or more times per week)

22b. How many alcohol units do you consume on a "typical" day when drinking alcohol?
One unit of alcohol is equivalent to one small bottle of beer, one glass of wine, or one mixed drink:

- 1-2
- 3-4
- 5-6
- 7-9
- 10 or more

22c. How often do you consume six or more alcohol units on a single occasion?
One unit of alcohol is equivalent to one small bottle of beer, one glass of wine, or one mixed drink:

- Never
- Less than monthly
- Monthly
- Weekly
- Daily or almost daily

Injuries

The following question pertains to new injuries during the specified period, not the treatment of old injuries. "Seeking medical attention" includes visits to a general practitioner, emergency room, or specialist healthcare services.

23. In the past 12 months, have you experienced one or more injuries that led you to seek medical or dental attention?

- Yes, one
- Yes, multiple
- No

(The following items (23a,b and c) appear if either of the following options is selected for question "23. In the past 12 months, have you experienced one or more injuries that led you to seek medical or dental attention?": Yes, one; Yes, multiple)

23a. What was the cause of the injury? (If more than one injury, consider the most severe)

- Accident
- Violence/assault
- Other cause

23b. Where did the injury occur?

- Road, street, sidewalk, pedestrian/cycle path - traffic accident (moving vehicle, including bicycle accidents)
- Road, street, sidewalk, pedestrian/cycle path - non-traffic accident
- Inside or outside a drinking establishment (bar, pub, restaurant)
- Residential area, indoors
- Residential area, outdoors
- Industrial or workshop area (factory, construction site, logging, farm)

- Institution, hospital, nursing home
- Sports or athletic area
- Natural environment - wilderness, sea, lake, river
- Other location of injury

23c. What were you doing when the injury occurred?

- At work
- Education
- Sports, exercise, training
- Outdoor activities, hunting, fishing
- Housework, gardening
- Play, hobby
- Other activity
- Noise

24. In the past 12 months, how bothered are you by noise from road traffic when you are at home?

- Not bothered
- Slightly bothered
- Moderately bothered
- Bothered a lot
- Extremely bothered

25. In the past 12 months, how bothered are you by noise from other sources when you are at home?

- Not bothered
- Slightly bothered
- Moderately bothered
- Bothered a lot
- Extremely bothered

Part 6: Quality of life

Now we have a series of questions about quality of life. You may find that some of the questions have already been asked earlier. However, it is important that you take the time to answer these questions as well.

26. Overall, how satisfied are you with your life at the moment?
    Please rate your answer on a scale from 0 to 10, where 0 means not satisfied at all and 10 means extremely satisfied.

27. Overall, to what extent do you feel that what you do in life is meaningful?
    Please rate your answer on a scale from 0 to 10, where 0 means not meaningful at all and 10 means extremely meaningful.

28. Think about how you have felt in the past 7 days. To what extent were you...?
    Please rate your answer on a scale from 0 to 10, where 0 means you did not experience the feeling at all and 10 means you experienced the feeling to a very large extent.

28.1.
0 - Not happy at all

1
2
3
4
5
6
7
8
9
10 - Very happy

28.2.
0 - Not worried at all
1
2
3
4
5
6
7
8
9
10 - Very worried

28.3.
0 - Not down or sad at all
1
2
3
4
5
6
7
8
9
10 - Very down or sad

28.4.
0 - Not irritated at all
1
2
3
4
5
6
7
8
9
10 - Very irritated

28.5.
0 - Not lonely at all
1
2
3
4
5
6
7
8
9
10 - Very lonely

28.6.
0 - Not engaged at all
1
2
3
4
5
6
7
8
9
10 - Very engaged

28.7.
0 - Not calm and relaxed at all
1
2
3
4
5
6
7
8
9
10 - Very calm and relaxed

28.8.
0 - Ikke engstelig i det hele tatt
1
2
3
4
5
6
7
8
9
10 - Svært engstelig

29. How much do you agree with the following statements?
Please rate your answer on a scale from 0 to 10, where 0 means completely disagree and 10 means completely agree.

29.1. My social relationships are supportive and rewarding:

29.2. I actively contribute to the happiness and quality of life of others:

In the past 14 days, how often have you experienced the following problems?

30. Little interest or pleasure in doing things
    - Not at all
    - Some days
    - More than half the days
    - Nearly every day or every day
31. Feeling down, depressed, or hopeless
    - Not at all
    - Some days
    - More than half the days
    - Nearly every day or every day

Social network and support

32. How often do you spend time with close friends?
    Do not count members of your own family.

    - Nearly every day
    - About once a week, but not every day
    - About once a month, but not every week
    - A few times a year
    - Less than once a year
    - I don't have close friends

33. Would you generally say that most people can be trusted, or that you can't be too careful when dealing with others?
    Please rate your answer on a scale from 0 to 10, where 0 means you can't be too careful and 10 means most people can be trusted.

34. To what extent do you feel that you belong in the place where you live?
    Please rate your answer on a scale from 0 to 10, where 0 means you don't feel any sense of belonging and 10 means you feel a strong sense of belonging.

35. Overall, how safe do you feel when you are out walking in your local area?
    Please rate your answer on a scale from 0 to 10, where 0 means you don't feel safe at all and 10 means you feel very safe.

Part 7. DEMOGRAPHIC INFORMATION

36. What is your highest completed education?
    - Primary school (up to 10 years)
    - Secondary education (minimum 3 years)
    - College/university (less than 4 years)
    - College/university (4 years or more)
37. If you live alone: Think about your total income. If you live with others, think about the total household income. How easy or difficult is it for you to make ends meet with this income?

    - Very difficult
    - Difficult
    - Somewhat difficult
    - Somewhat easy
    - Easy
    - Very easy
    - Don't know

38. What is your current work or life situation?
    Check one or more options

    - Full-time employment (32 hours or more per week)
    - Part-time employment (less than 32 hours per week)
    - Self-employed
    - On sick leave
    - Unemployed
    - Receiving disability benefits or vocational rehabilitation
    - Receiving social assistance
    - Retired or receiving early retirement benefits
    - Student or pupil
    - Military service, civilian service
    - Homemaker

39. Are you married/cohabiting, single, or do you have a partner?
    - Married/registered partner
    - Cohabiting
    - Have a partner (not living together)
    - Single

ABOUT FAMILY (LIVING CONDITIONS)

Upbringing, when you were 0-18 years old.

40. Who did you grow up with?
    Check one or more options

    - Mother
    - Stepmother
    - Father
    - Stepfather
    - Foster/adoptive parents
    - Siblings

- Other children under 18
- Other adults

41. Did your parents get divorced or separate when you were a child?
    - No
    - Yes, before I was 7 years old
    - Yes, when I was 7-18 years old
42. Was there a lot of arguing, unrest, conflicts, or difficult communication in your childhood home?
    - To a very high extent
    - To a high extent
    - To a small extent
    - To a very small extent
    - Not at all

43. Could you seek support from an adult you felt safe with during your upbringing?
    - To a very high extent
    - To a high extent
    - To a small extent
    - To a very small extent
    - Not at all

44. Do you struggle with painful memories from your upbringing due to loss, betrayal, neglect, violence, abuse, or mistreatment?
    - To a very high extent
    - To a high extent
    - To a small extent
    - To a very small extent
    - Not at all
45. When you think about your childhood/upbringing, how would you describe it?
    - Very good
    - Good
    - Average
    - Difficult
    - Very difficult

ABOUT PARENTS' ALCOHOL USE

Think back to your upbringing. Choose the answer option that best describes your feelings and experiences regarding your parents' alcohol habits.
This includes step-parents or other guardians. Take your time and answer as accurately as possible.

46. Have you ever thought that one of your parents (biological or step-parents) had an alcohol problem?
    - Yes
    - No
47. Have you ever encouraged one of your parents to stop drinking alcohol?
    - Yes
    - No

48. Have you ever argued with a parent because they were drinking alcohol?

- Yes
- No

49. Have you ever heard your parents argue when one of them was drunk?
    - Yes
    - No

50. Have you ever wanted to hide or empty one of your parents' bottles of alcohol?
    - Yes
    - No

51. Have you ever wished that a parent would stop drinking alcohol?
    - Yes
    - No
